# Acquired Tracheoesophageal Fistula After Esophageal Atresia Repair

**DOI:** 10.4274/balkanmedj.galenos.2019.2019.8.60

**Published:** 2019-12-20

**Authors:** Özlem Boybeyi Türer, Feridun Cahit Tanyel, Tutku Soyer

**Affiliations:** 1 Department of Pediatric Surgery, Hacettepe University School of Medicine, Ankara, Turkey

**Keywords:** Children, congenital anomaly, diagnosis, surgery, esophageal atresia, tracheoesophageal fistula

## Abstract

**Background:**

Recurrence of tracheoesophageal fistula is a frequent complication after esophageal atresia repair. Acquired tracheoesophageal fistulas are long new fistulas that are localized at sites that are not typical of the congenital tracheoesophageal fistula. We present four cases to discuss the diagnostic and management challenges concerning various acquired tracheoesophageal fistula localizations.

**Case Report:**

We retrospectively evaluated the medical records of patients admitted with acquired tracheoesophageal fistula in the last 5 years. Among the 16 postoperative tracheoesophageal fistulas, 4 were classified as acquired tracheoesophageal fistula. Patients’ admission age ranged from 1 to 8 years. The female to male ratio was 2:2. The presented cases were admitted with recurrent respiratory tract infections, choking, and coughing. The acquired tracheoesophageal fistulas were observed between the esophagus and cervical trachea, between the esophagus and the right bronchus passing through intrathoracic abscess cavity, in the right bronchus, and between the colon conduit and trachea. One of the acquired tracheoesophageal fistulas healed spontaneously, whereas others required surgical ligation.

**Conclusion:**

Acquired tracheoesophageal fistula most often occurs secondary to local or diffuse mediastinitis. Acquired tracheoesophageal fistula may appear at unusual sites not typical of congenital tracheoesophageal fistula, such as esophagus-to-right bronchus and conduit to trachea. Therefore, the unusual locations of acquired tracheoesophageal fistula should be borne in mind, and patients evaluated and managed more comprehensively.

Recurrence of tracheoesophageal fistula (TEF) is observed in up to 5% to 10% of esophageal atresia cases (1). Smithers et al. (2) classified TEF as congenital, recurrent, and acquired based on their etiology and anatomy. TEFs persisting after the operation is considered as congenital TEF, either missed during initial operation or incompletely repaired. Recurrent TEFs are fistulas that occur after the initial successful repair in the same location as the index fistula. Acquired TEFs (acq-TEF) are new pathways between the airway and esophagus and appear at locations not typical for a fistula (2). They include communications between esophageal anastomosis and pulmonary parenchyma, bronchus, or trachea. Moreover, they can be observed, in rare instances, between the colon and gastric conduit along with the entire respiratory system. Acq-TEFs are difficult to diagnose and pose a significant treatment challenge. Therefore, we present four cases to discuss the challenges in the diagnosis and treatment of acq-TEF.

## CASE PRESENTATION

This study was performed in accordance with the Declaration of Helsinki and was approved by the Local Ethical Committee. Informed consent was obtained from all cases. We retrospectively evaluated the medical records of patients admitted with postoperative TEF between 2013 and 2019. Overall, 42 patients were operated for primary esophageal atresia, and 16 patients were managed for postoperative TEF between 2013 and 2019. Among the 16 postoperative TEF cases, 11 were recurrence, 1 was congenital (missed in the initial operation), and 4 (25%) were acquired. The primary repair of two cases with recurrent TEF and one case with acquired TEF (case 3) was performed at our center. The primary repair of the remaining 13 postoperative TEFs was performed at another center.

**Figure 1:**
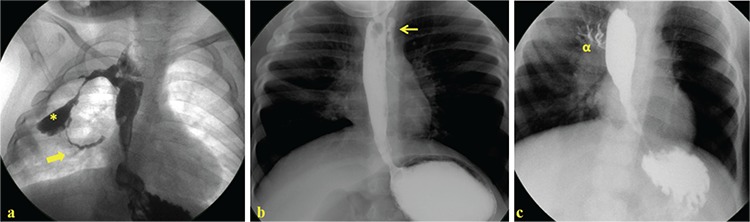
(a) The esophagography of Case 1 revealing acquired fistula (arrow) between the esophagus and right bronchus passing through the abscess pouch (*), (b) The esophagography of case 2 revealing acquired fistula (arrow) between the cervical esophagus and trachea, (c). The esophagography of case 4 revealing acquired fistula between the esophagus and right main bronchus (α)

The demographics and previous medical histories of the presented cases are given in Table 1. The admission complaints, clinical features, and management details are presented in Table 2 In all cases, esophagography was performed, and TEFs localized at an unusual site unlike a typical TEF were diagnosed as acq-TEF. The acq-TEFs were observed between the esophagus and cervical trachea, between the esophagus and right bronchus passing through the intrathoracic abscess cavity, in the right bronchus, and between the colon conduit and trachea (Figure 1).

Case 1 was admitted with coughing during feeding and respiratory tract infections lasting for 2 months when she was 3 years old. Because of the poor general condition of the case, we decided to manage the patient conservatively. During this period, oral feeding was ceased, and tube feeding and broad-spectrum antibiotics were recommended. We observed spontaneous healing of the fistula after 2 months. She has been on full oral feeding for the last 1 year without any respiratory symptoms.

Case 2 was admitted with coughing with liquids lasting for 3 months. We detected acq-TEFs between the esophagus and cervical trachea and repaired it through a cervical incision. The patient has had an uneventful follow-up for the last 3 years.

Case 3 underwent esophageal replacement with a colon conduit supplied by the right middle colic artery at the age of 1 year. She had leakage from the colon-esophageal anastomosis for 3 weeks. We detected TEF between the cervical trachea and the colon conduit. We performed a cervical esophagostomy and ligation of the fistula between the colon conduit and trachea through a cervical incision. Muscle graft was laid on the sutures. Three months after the operation, she had a redo colon-esophageal anastomosis through a median sternotomy. She has been followed up uneventfully for the last 6 months.

Case 4 was admitted with wheezing and coughing for 4 months. The upper GI study revealed TEF to the right main bronchus. A right posterolateral thoracotomy was performed to repair the fistula. The patient’s postoperative contrast study was normal, and he has been on full oral feeding for the last 4 years.

## DISCUSSION

Postoperative TEF after esophageal atresia repair is both a diagnostic and surgical challenge. In most of the large series, all postoperative TEFs were defined as “recurrent” TEFs, and little was known regarding the acquired fistulas. Based on the classification of postoperative TEFs by Smithers et al. (2), acq-TEFs are defined as new communications between the esophagus and airway. The most common risk factors in the etiology of TEF recurrence are anastomotic leaks and strictures (3,4,5). Although balloon dilation is a safe procedure in the treatment of strictures, it is considered a risk factor for TEF development (6). In recurrent TEFs, anastomotic leaks cause recanalization of the previous pathway. However, acq-TEFs are the result of new pathways that occur because of inflammation and mediastinitis. Among our patients, three had prolonged leaks and mediastinitis.

Notably, diagnosis and localization of an acq-TEF is challenging. Nonetheless, a missed congenital TEF should be excluded. The new fistulas can be between the esophageal anastomosis and pulmonary parenchyma, bronchus, the trachea, colon, or gastric conduit. For diagnosis, contrast esophagography in a prone position is a reliable method and has a success rate of 94% in detecting the fistula (7,8). Furthermore, bronchoscopy and catheter insertion through the fistula is recommended to confirm the diagnosis and localize the fistula preoperatively. However, we could not insert a catheter through the fistula in our study cases because of the abnormal localization of the fistulas, which is the primary reason for making the diagnosis and surgical management challenging.

The surgical treatment of recurrent and acq-TEFs is comparatively difficult and hazardous than congenital ones because of dense adhesions and mediastinal fibrosis. Therefore, failure rates are common, and several repairs may be needed. Notably, no consensus exists regarding the best time and type of surgical repair in recurrent TEFs. The timing of the operation typically depends on the general condition of the patient. Therefore, during prolonged waiting periods, the child can be placed on tube feeding. Despite no consensus, it is typically recommended to wait at least 5-6 weeks for the resolution of the inflammation (3). We observed spontaneous healing of the acq-TEF during the waiting period in our case 1. To the best of our knowledge, spontaneous healing of an acq-TEF has not been reported until date. Because acq-TEFs occur after severe inflammation owing to leaks, scarring of the mediastinal structures may close the new pathway between the esophagus and airway. Even though it is not possible to make a firm conclusion based on just one case, the possibility of spontaneous healing of fistula can be borne in mind in such cases. In addition, we recommend waiting only until the inflammation resolves, and the general condition of the patient becomes suitable for operation. During the waiting period, tube feeding and antibiotic therapy might be pursued. Moreover, repeat esophagography may be performed before the surgery in case of a prolonged waiting period.

The surgical access for TEF repair depends on the location of the fistula. A cervical incision can be used for TEFs localized between the cervical trachea and the esophagus. Thoracotomy is required in TEFs between the thoracic esophagus and bronchus or lung parenchyma. Notably, tissue interposition between suture lines is mandatory to avoid recurrence. Pleural, pericardial, and muscle flaps can be used. We also interposed tissue between the suture lines in all our cases.

In conclusion, acq-TEF most often occurs secondary to local or diffuse mediastinitis. Acq-TEF may occur at unusual locations leading to both diagnostic and surgical challenges. Nonetheless, the possibility of spontaneous healing should be borne in mind in cases with acq-TEFs.

## Figures and Tables

**Table 1:**
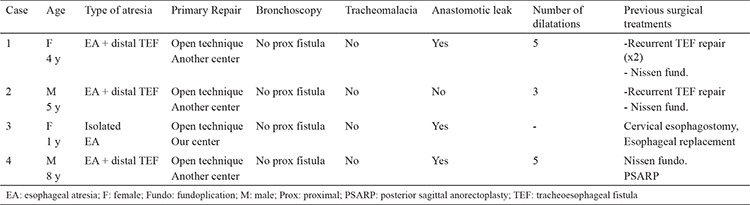
Demographic features and previous medical history of the cases with acq-TEF

**Table 2:**
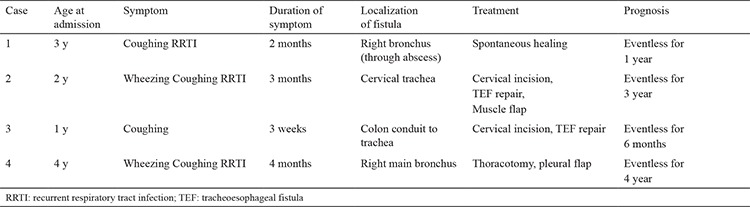
The admission complaints, clinical features, and management details of the cases.
